# Hydrogen production by the hyperthermophilic bacterium *Thermotoga maritima* Part II: modeling and experimental approaches for hydrogen production

**DOI:** 10.1186/s13068-016-0681-0

**Published:** 2016-12-19

**Authors:** Richard Auria, Céline Boileau, Sylvain Davidson, Laurence Casalot, Pierre Christen, Pierre Pol Liebgott, Yannick Combet-Blanc

**Affiliations:** Aix Marseille Université, Université de Toulon, CNRS, IRD, MIO UM 110, Mediterranean Institute of Oceanography, 13288 Marseille, France

**Keywords:** *Thermotoga maritima*, Hyperthermophile, Hydrogen, Modeling, Inhibition

## Abstract

**Background:**

*Thermotoga maritima* is a hyperthermophilic bacterium known to produce hydrogen from a large variety of substrates. The aim of the present study is to propose a mathematical model incorporating kinetics of growth, consumption of substrates, product formations, and inhibition by hydrogen in order to predict hydrogen production depending on defined culture conditions.

**Results:**

Our mathematical model, incorporating data concerning growth, substrates, and products, was developed to predict hydrogen production from batch fermentations of the hyperthermophilic bacterium, *T. maritima*. It includes the inhibition by hydrogen and the liquid-to-gas mass transfer of H_2_, CO_2_, and H_2_S. Most kinetic parameters of the model were obtained from batch experiments without any fitting. The mathematical model is adequate for glucose, yeast extract, and thiosulfate concentrations ranging from 2.5 to 20 mmol/L, 0.2–0.5 g/L, or 0.01–0.06 mmol/L, respectively, corresponding to one of these compounds being the growth-limiting factor of *T. maritima*. When glucose, yeast extract, and thiosulfate concentrations are all higher than these ranges, the model overestimates all the variables. In the window of the model validity, predictions of the model show that the combination of both variables (increase in limiting factor concentration and in inlet gas stream) leads up to a twofold increase of the maximum H_2_-specific productivity with the lowest inhibition.

**Conclusions:**

A mathematical model predicting H_2_ production in *T. maritima* was successfully designed and confirmed in this study. However, it shows the limit of validity of such mathematical models. Their limit of applicability must take into account the range of validity in which the parameters were established.

**Electronic supplementary material:**

The online version of this article (doi:10.1186/s13068-016-0681-0) contains supplementary material, which is available to authorized users.

## Background

Because of the increasing demand for energy, due to economic and population rapid growths and because of the damaging effect of the fossil energies on the environment (global warming), governments are focusing on alternative energy sources for fuels. In response to this dual problem (depletion and pollution), the development of a new, more environmentally friendly, and healthy energy is necessary.

Presently, the development of biofuels from renewable plant biomass is a way to reduce fossil fuel consumption. Among the potential biofuels, hydrogen appears as one of the energy sources for the future. Indeed, hydrogen is highly reactive, with high energy density (122 MJ/kg, compared to 50.1 MJ/kg for methane, 29.7 MJ/kg for ethanol, and 47.3 MJ/kg for gasoline) and is directly convertible into electricity with high efficiency (>80%). In addition, it is a low-carbon fuel which combustion produces only water, making it an excellent candidate in terms of environmental impact. Overall, the use of hydrogen shows a 10% growth per year, leading to represent 8–10% of total energy in 2025.

Currently, hydrogen production is closely dependent on fossil fuels (natural gas and hydrocarbons). However, new approaches for hydrogen production, such as biophotolysis, photofermentation, and dark fermentation, offer less costly technological solutions in terms of energy balance and are friendlier to the environment. Among these techniques, dark fermentation is of great interest because it allows the biodegradation of complex residues using a broad spectrum of microorganisms and enzymes. In addition, it is performed with abundant, inexpensive, renewable, and biodegradable agricultural waste [1].

To be economically viable, one of the main challenges of dark fermentation is to achieve both high hydrogen productivity and yield. Hydrogen is produced by both mesophile and (hyper) thermophile anaerobic bacteria. In general, the latter ones show slightly lower hydrogen production rates but higher yields. The elevated temperature (70–110 °C) has several advantages by reducing (1) the hydrogen solubility which is known to be a strong inhibitor of growth [2–4], (2) the variety in fermentation by-products [5] and (3) the sensitivity to contamination by H_2_-consumer and pathogen bacteria present in the waste. Moreover, the high temperature promotes the enzymatic hydrolysis of a wide range of carbohydrates (starch, cellulose, hemicelluloses…) [6–9]. Among the hyperthermophilic anaerobic hydrogen-producing bacteria (>80 °C), *Thermotogales* were one of the most studied [6, 10–12]. Several *Thermotoga* sp. (*neapolitana*, *maritima*, *elfii*, *petrophila*, *naphtophila*,…) metabolized glucose with high hydrogen yields of 3–4 mol/mol [6, 12–16]. Maximal hydrogen productivity of some *Thermotoga* strains was reported between 2.7 and 12.4 mmol/L h [6, 14, 16, 17]. Higher hydrogen productivity requires determining the conditions influencing the growth and the metabolism of the *Thermotogales*. Among them, optimal concentrations of glucose, yeast, sulfur, dissolved hydrogen, carbon dioxide, etc., have to be established [6, 16, 17]. Most *Thermotoga* species have been reported to reduce elemental sulfur to H_2_S [18, 19]. Huber et al. [20] proposed that the addition of elemental sulfur stimulates the growth of *T. maritima* on glucose by reducing the inhibitory effect of hydrogen. Schroder et al. [6] supported this hypothesis by showing that, on glucose, sulfur reduction to H_2_S stimulated *T. maritima* growth. On the contrary, Boileau et al. [part I, 21] showed that the addition of small amounts of thiosulfate, as well as some other sulfur sources, allowed a significant increase of *T. maritima* growth and its hydrogen production. These authors confirmed that the sulfur compound was not used in a detoxification process but rather was assimilated by *T. maritima* leading to an increase in biomass, and therefore in the amount of hydrogen produced.

To improve the understanding of the biological and physical mechanisms that govern the hydrogen production by dark fermentation, mathematical modeling can be an appropriate tool. The aim of the present study is to propose a mathematical model incorporating kinetics of growth, consumption of substrates, product formations, and inhibition by hydrogen in order to predict hydrogen production depending on defined culture conditions. To the best of our knowledge, the most comprehensive kinetic model predicting the hydrogen production by a thermophilic bacterium was proposed by Ljunggren et al. [3]. This kinetic model takes into account the microbial growth of the extreme thermophilic *Caldicellulosiruptor saccharolyticus*, its substrate consumption and product formations and, the liquid-to-gas mass transfer. This model predicted high oversaturation of hydrogen in the liquid (12–34 times the equilibrium concentration) comparable to the experimentally obtained values. The authors have shown that the dissolved hydrogen concentration was a function of the stripping rate and the hydrogen productivity. In the present study, a mathematical model was developed to predict hydrogen production from batch fermentations of the hyperthermophilic bacterium, *T. maritima*. This model incorporates the kinetics of growth, consumptions of substrates (glucose, yeast, and thiosulfate), and product formations (H_2_, CO_2_, H_2_S, acetate, and lactate). It includes the inhibition by hydrogen and the liquid-to-gas mass transfer of H_2_, CO_2_, and H_2_S. Most kinetic parameters of the model were obtained from batch experiments without any fitting. The limits of its applicability were clearly established.

## Methods

### Strain and culture medium

*Thermotoga maritima* strain MSB8 (DSMZ 3109) was cultivated as previously described [part I, 21]. Basal medium containing, per liter: NH_4_Cl 0.5 g, K_2_HPO_4_ 0.3 g, 0.3 g, CaCl_2_ 0.1 g, KCl 0.1 g, NaCl 20 g, MgCl_2_ 0.2 g, yeast extract 1.0 g, and glucose 20 mmol/L was used. Balch trace mineral element solution (10 mL) was added [part I, 21]. The inoculum was obtained from three bottles of 100 mL each, containing 50 mL of liquid culture.

### Experimental system, operating conditions, and analytical methods

Experimental system, operating conditions, and analytical methods were specified [part I, 21]. *T. maritima* was batch cultivated in a similar 2-L double-jacket glass bioreactor (FairMenTec, France) with a 1.5-L working volume. The temperature was maintained constant at 80 ± 1 °C and pH was controlled at 7 ± 0.1 by the addition of sodium hydroxide (NaOH 0.5 mmol/L). The inlet gas stream of N_2_ was controlled via a mass-flow meter (Bronkhorst, range 0–500 SCCM), and the one composed of the mixture of N_2_ and H_2_ was prepared using two mass-flow meters (Bronkhorst, range 0–500 SCCM and 0–100 SCCM, The Netherlands). The stirring was set to 350 rpm. The online measurements of CO_2_, H_2_, and H_2_S concentrations, bioreactor liquid volume, and NaOH consumption are described [part I, 21].

For each experiment, three successive batches were carried out. The first batch was always considered as an adaptation batch, the following two being in general well reproducible.

OD (Optical Density), hydrogen, glucose, acetate, lactate, and hydrogen sulfide concentrations were determined as previously described [part I, 21]. Cell dry weight was calculated from OD data using the same relation of 1 OD unit = 330 mg/L.

Thiosulfate concentration was quantified by ion chromatography (761 Compact IC Metrohm, Metrohm, Villebon-sur-Yvette, France) equipped with a Metrosep Anion Supp1 column (Metrohm).

### Mathematical model

The model developed in this study incorporates the kinetics of growth of *T. maritima*, glucose, yeast extract, and thiosulfate consumptions and product, such as hydrogen (H_2_), carbon dioxide (CO_2_), hydrogen sulfide (H_2_S), acetate, and lactate formations. It takes into account the transfer of H_2_, CO_2_, and H_2_S, as well as the chemical equilibrium between CO_2_ and bicarbonates (HCO_3_^−^) and between H_2_S and hydrosulfide ions (HS^−^).

For the operational parameters of this study, the main end products, resulting from the anaerobic fermentation of glucose by *T. maritima,* are acetate, lactate, H_2_, CO_2_, biomass, and EPS (Extracellular polysaccharides) [22]. In this case, the following biochemical reaction occurring during the fermentation is:

1 $${\text{C}}_{6} {\text{H}}_{12} {\text{O}}_{6} + k\;{\text{H}}_{2} O \to a\;{\text{C}}_{2} {\text{H}}_{4} {\text{O}}_{2} + b\;{\text{C}}_{3} {\text{H}}_{6} {\text{O}}_{3} + 2a {\text{H}}_{2} + a\;{\text{CO}}_{{\begin{array}{*{20}c} 2 \\ \end{array} }} + c\;{\text{Biomass}} + d {\text{EPS}}$$

The value of the stoichiometric parameters *a*, *b*, *c,* and *d* in the Eq. 1 are unknown. The parameter *a* was estimated using a methodology described in the Determination of kinetic and mass transfer parameters section. Glucose fermentation in *T. maritima,* under controlled physicochemical conditions, shows that about 5 and 22% of the consumed glucose are converted into biomass and EPS, respectively [22]. These two values of percentages were used to evaluate *c* and *d* parameters. The stoichiometric parameter *b* relates to the lactate production and was determined by the difference between the amount of carbon from the consumed glucose and the amount of carbon found in all the end products: acetate, carbon dioxide, biomass, and EPS.

At low concentration, thiosulfate is a sulfured nutriment for *T. maritima* growth used for the synthesis of cellular materials [part I, 21]. Thus, this sulfur is incorporated to the biomass according to the elemental composition of *T. maritima* $({\text{CH}}_{1.6} {\text{O}}_{0.6} {\text{N}}_{0.2} {\text{S}}_{0.005} )$ determined by Rinker and Kelly [23].

The low amount of remaining sulfur leads to the production of hydrogen sulfide as follows:

2 $$S_{2} {\text{O}}_{3}^{2 - } + 4 {\text{H}}_{2} \to 2 {\text{H}}_{2} {\text{S}} + {\text{H}}_{2} {\text{O}} + 2 {\text{OH}}^{ - }$$

#### Growth kinetics, substrate, and nutrient consumptions

The specific growth rate ($\mu$) is given by Monod kinetics. It depends on the glucose (Glu), yeast extract (Yeast), thiosulfate (Thio), dissolved-H_2_ ([H_2_]) concentrations and the equivalent concentrations of glucose ($\varepsilon_{1}$) and thiosulfate ($\varepsilon_{2}$) in the yeast extract. Although *T. maritima* can grow (slightly) without yeast extract [part I, 21], we consider that yeast extract is essential for the growth as a first approximation. The mass balance for *T. maritima* growth is described by the following expression:

3 $$\frac{{{\text{d}}X}}{{{\text{d}}t}} = (\mu - \mu_{\text{d}} ) X$$

4 $$\mu = \mu_{max } \left( {\frac{{Glu + \varepsilon_{1} }}{{\left( {Glu + \varepsilon_{1} } \right) + K_{sglu} }}} \right)\left( {\frac{Yeast }{{ Yeast + K_{syeast} }}} \right)\left( {\frac{{Thio + \varepsilon_{2} }}{{\left( {Thio + \varepsilon_{2} } \right) + K_{sthio} }}} \right)\left( {1 - \left( {\frac{{\left[ {H_{2} } \right]}}{{\left[ {H_{2crit} } \right]}}} \right)^{N} } \right),$$

where *X* and *μ*_max_ are the cell mass concentration and the maximum specific growth, respectively. $\mu_{\text{d}}$ is the cell death rate. [H_2crit_] is the critical dissolved-H_2_ concentration for which inhibition is 100%. *N* is the exponential parameter describing the level of inhibition. $K_{\text{sglu}} , K_{\text{syeast}}$, $K_{\text{sthio}}$ are the saturation constants of glucose, yeast extract, and thiosulfate.

The mass balance of glucose (Eq. 5), yeast extract (Eq. 6), and thiosulfate (Eq. 7) can be written as follows:

5 $$\frac{{{\text{d}}({\text{Glu}} + \varepsilon_{1} )}}{{{\text{d}}t }} = - \left( {\frac{\mu }{{Y_{{{\text{X}}/{\text{GLU}}}} }} + m_{\text{GLU}} } \right) X$$

6 $$\frac{\text{dYeast}}{{{\text{d}}t }} = - \frac{\mu X}{{Y_{{{\text{X}}/{\text{YEAST}}}} }}$$

7 $$\frac{{{\text{d}}({\text{Thio}} + \varepsilon_{2} )}}{{{\text{d}}t }} = - \frac{\mu X}{{Y_{{{\text{X}}/{\text{THIO}} }} }}$$

8 $${\text{with}}:Y_{{{\text{X}}/{\text{THIO}} }} = Y_{{{\text{X}}/{\text{THIO}}}}^{\text{X}} + Y_{{{\text{X}}/{\text{THIO}}}}^{{{\text{H}}_{2} {\text{S}}}}$$

$Y_{{{\text{X}}/{\text{GLU}}}}$, $Y_{{{\text{X}}/{\text{YEAST}}}}$ and $Y_{{{\text{X}}/{\text{THIO}}}}$ are the yields of biomass on glucose, yeast extract, and thiosulfate, respectively. $m_{\text{GLU}}$ is the maintenance coefficient [ratio between the consumption rate of glucose and maximum biomass determined at the end of the growth when the specific growth rate (*μ*) is close to 0]. $Y_{{{\text{X}}/{\text{THIO}}}}$ is the sum of two yields: the equivalent sulfur from thiosulfate incorporated into the biomass ($Y_{{{\text{X}}/{\text{THIO}}}}^{\text{X}} )$ and the equivalent sulfur from thiosulfate released as H_2_S $\left( {Y_{{{\text{X}}/{\text{THIO}}}}^{{{\text{H}}_{2} {\text{S}}}} } \right)$.

#### Product formation in the liquid phase

The products formed during the fermentation are acetate, lactate, hydrogen, carbon dioxide, and hydrogen sulfide. The product formation rates are expressed using the following equations:

9 $$\frac{\text{dAct}}{{{\text{d}}t}} = Y_{{{\text{ACT}}/{\text{GLU}}}} \left( {\frac{\mu }{{Y_{{{\text{X}}/{\text{GLU}}}} }} + m_{\text{GLU}} } \right)X$$

and Eq. 10 for the determination of lactate (obtained from the Eq. 1):

10 $$\frac{\text{dLact}}{{{\text{d}}t}} = \frac{{{\text{d}}\left( {{\text{Glu}} + \varepsilon_{1} } \right)}}{{{\text{d}}t }} (6 Y_{{{\text{ACT}}/{\text{GLU}}}} - 1.62) - 2 \frac{\text{dAct}}{{{\text{d}}t}} - \frac{{{\text{d}}\left[ {{\text{CO}}_{2} } \right]}}{{{\text{d}}t}}$$

11 $$\frac{{{\text{d}}\left[ {{\text{H}}_{2} } \right]}}{{{\text{d}}t}} = - {\text{KlaH}}_{2} \left( {\left[ {{\text{H}}_{2} } \right] - \left[ {{\text{H}}_{2} } \right]^{*} } \right) + Y_{{{\text{H}}_{2} /{\text{GLU}}}} \left( {\frac{\mu }{{Y_{{{\text{X}}/{\text{GLU}}}} }} + m_{\text{GLU}} } \right)X - \frac{{Y_{{{\text{H}}_{2} {\text{S}}/{\text{THIO}}}} Y_{{{\text{H}}_{2} /{\text{H}}_{2} {\text{S}}}} }}{{Y_{{{\text{X}}/{\text{THIO}}}}^{{{\text{H}}_{2} {\text{S}}}} }} \mu X$$

12 $$\frac{{{\text{d}}\left[ {{\text{CO}}_{2} } \right]}}{{{\text{d}}t}} = - {\text{KlaCO}}_{2} \left( {\left[ {{\text{CO}}_{2} } \right] - \left[ {{\text{CO}}_{2} } \right]^{*} } \right) + Y_{{{\text{CO}}_{2} /{\text{GLU}}}} \left( {\frac{\mu }{{Y_{{{\text{X}}/{\text{GLU}}}} }} + m_{\text{GLU}} } \right) X - \frac{{K_{1 } }}{{ 10^{{ - {\text{pH}}}} }}\left[ {{\text{CO}}_{2} } \right] + \left[ {{\text{HCO}}_{3}^{ - } } \right]$$

13 $$\frac{{{\text{d}}\left[ {{\text{H}}_{2} {\text{S}}} \right]}}{{{\text{d}}t}} = - {\text{KlaH}}_{2} {\text{S }}\left( {\left[ {{\text{H}}_{2} {\text{S}}} \right] - \left[ {{\text{H}}_{2} {\text{S}}} \right]^{ *} } \right) + \frac{{Y_{{{\text{H}}_{2} {\text{S}}/{\text{THIO}}}} }}{{Y_{{{\text{X}}/{\text{THIO}}}}^{{{\text{H}}_{2} {\text{S}}}} }}\mu X - \frac{{K_{2 } }}{{ 10^{{ - {\text{pH}}}} }}\left[ {{\text{H}}_{2} {\text{S}}} \right] + \left[ {{\text{HS}}^{ - } } \right]$$

14 $$\frac{{{\text{d}}\left[ {{\text{HCO}}_{3}^{ - } } \right]}}{{{\text{d}}t}} = \frac{{K_{1 } }}{{ 10^{{ - {\text{pH}}}} }}\left[ {{\text{CO}}_{2} } \right] - \left[ {{\text{HCO}}_{3}^{ - } } \right]$$

15 $$\frac{{{\text{d}}\left[ {{\text{HS}}^{ - } } \right]}}{{{\text{d}}t}} = \frac{{K_{2 } }}{{ 10^{{ - {\text{pH}}}} }}\left[ {{\text{H}}_{2} {\text{S}}} \right] - \left[ {{\text{HS}}^{ - } } \right]$$

${\text{Act}},\;{\text{Lact}},\; \left[ {{\text{H}}_{2} } \right],\; \left[ {{\text{CO}}_{2} } \right],\; \left[ {{\text{H}}_{2} {\text{S}}} \right], \left[ {{\text{HCO}}_{3}^{ - } } \right] \;{\text{and}},\; \left[ {{\text{HS}}^{ - } } \right]$ are the concentrations in the liquid phase of acetate, lactate, hydrogen, carbon dioxide, hydrogen sulfide, bicarbonate, and bisulfide ions, respectively. $\left[ {{\text{H}}_{2} } \right]^{*} , \;\left[ {{\text{CO}}_{2} } \right]^{*} \;{\text{and}},\; \left[ {{\text{H}}_{2} {\text{S}}} \right]^{ *}$ are the dissolved concentrations of these compounds at equilibrium. $Y_{{{\text{ACT}}/{\text{GLU}}}}$,$Y_{{{\text{H}}_{2} /{\text{GLU}}}} , Y_{{{\text{CO}}_{2} /{\text{GLU}}}}$ are the yields of acetate, H_2_ and CO_2_. $Y_{{{\text{H}}_{2} /{\text{H}}_{2} {\text{S}}}} \;{\text{and}}\;Y_{{{\text{H}}_{2} {\text{S}}/{\text{THIO}}}}$ are the stoichiometric H_2_ on H_2_S yield and the stoichiometric H_2_S on thiosulfate yield (Eq. 2), respectively.

${\text{KlaH}}_{2} , \;{\text{KlaCO}}_{2}, \;{\text{and}}\;{\text{KlaH}}_{2} {\text{S}}$ represent the volumetric mass transfer coefficients for H_2_, CO_2_, and H_2_S. *K*_1_ and *K*_2_ are the dissociation constants.

Here, H_2_ inhibits its own production and consequently its acetate production. Thereby, $Y_{{{\text{ACT}}/{\text{GLU}}}}$ can be written as follows [3]:

16 $$Y_{{{\text{ACT}}/{\text{GLU}}}} = Y_{{{\text{ACT}}/{\text{GLU}}}}^{ \rm{max} } \left( {1 - \left( {\frac{{\left[ {{\text{H}}_{2} } \right]}}{{\left[ {{\text{H}}_{{2{\text{crit}}}} } \right]}}} \right)^{N} } \right)$$

The maximum yield of acetate on glucose $Y_{{{\text{ACT}}/{\text{GLU}}}}^{ \rm{max} }$ was estimated from experiments (see Determination of kinetic and mass transfer parameters) for H_2_ percentage in the gas phase equal to 0. $Y_{{{\text{H}}_{2} /{\text{GLU}}}}$ and $Y_{{{\text{CO}}_{2} /{\text{GLU}}}}$ were deduced from $Y_{{{\text{ACT}}/{\text{GLU}}}}$ using the Eq. 1.

#### Mass balance in the gas phase

Hydrogen, carbon dioxide, and hydrogen sulfide are produced in the liquid phase and then transferred into the gas phase. The mass balance of these gaseous compounds can be expressed as follows:

17 $$\frac{{{\text{d}}H_{2} }}{{{\text{d}}t}} = \frac{{V_{\text{l}} }}{{V_{\text{g}} }}{\text{KlaH}}_{2} \left( {\left[ {{\text{H}}_{2} } \right] - \left[ {{\text{H}}_{2} } \right]^{*} } \right) - \frac{{Q_{\text{g}} }}{{V_{\text{g}} }} {\text{H}}_{2}$$

18 $$\frac{{{\text{dCO}}_{2} }}{{{\text{d}}t}} = \frac{{V_{\text{l}} }}{{V_{\text{g}} }}{\text{KlaCO}}_{2} \left( {\left[ {{\text{CO}}_{2} } \right] - \left[ {{\text{CO}}_{2} } \right]^{*} } \right) - \frac{{Q_{\text{g}} }}{{V_{\text{g}} }}{\text{CO}}_{2}$$

19 $$\frac{{{\text{dH}}_{2} {\text{S}}}}{{{\text{d}}t}} = \frac{{V_{\text{l}} }}{{V_{\text{g}} }}{\text{KlaH}}_{2} {\text{S }}\left( {\left[ {{\text{H}}_{2} {\text{S}}} \right] - \left[ {{\text{H}}_{2} {\text{S}}} \right]^{ *} } \right) - \frac{{Q_{\text{g}} }}{{V_{\text{g}} }}{\text{H}}_{2} {\text{S}}$$

20 $$Q_{\text{g}} = Q_{{N_{2} }} + Q_{{{\text{H}}_{2} }} + Q_{{{\text{CO}}_{2} }} + Q_{{{\text{H}}_{2} {\text{S}}}}$$

$Q_{\text{g}}$,$V_{\text{l}} \;{\text{and}},\;V_{\text{g}}$ are the outlet-gas flow rate, the liquid and free-gaseous-space volumes in the reactor, respectively. $Q_{{{\text{N}}_{2} }} ,\;Q_{{{\text{H}}_{2} }} ,\;Q_{{{\text{CO}}_{2} }} \;{\text{and}},\;Q_{{{\text{H}}_{2} {\text{S}}}}$ are the inlet gas (N_2_) flow rate, H_2_, CO_2_, and H_2_S gas flow rate productions, respectively.

#### Equilibrium constants and stoichiometric equations

The thermodynamic equilibrium of the dissolved compounds $\left[ {{\text{H}}_{2} } \right]$, $\left[ {{\text{CO}}_{2} } \right] \;{\text{and}},\;\left[ {{\text{H}}_{2} {\text{S}}} \right]$ is described by the Henry’s law:

21 $$\left[ {{\text{H}}_{2} } \right]^{*} = {\text{Kh}}_{2} R\;T\;{\text{H}}_{2}$$

22 $$\left[ {{\text{CO}}_{2} } \right]^{*} = {\text{K}}_{{{\text{CO}}_{2} }} R\;T {\text{CO}}_{2}$$

23 $$\left[ {{\text{H}}_{2} {\text{S}}} \right]^{*} = K{\text{h}}_{2} {\text{s}}\;R\;T\;{\text{H}}_{2} {\text{S}}$$

${\text{Km}}\;(m = {\text{h}}_{2} , {\text{co}}_{2} , {\text{h}}_{2 } {\text{s}})$ are the H_2_, CO_2_, and H_2_S Henry’s constants.

The dissociations of $\left[ {{\text{HCO}}_{3}^{ - } } \right]$ into $[{\text{CO}}_{3}^{2 - } ]$ and [${\text{HS}}^{ - } ]$ into $[{\text{S}}^{2 - } ]$ are considered negligible at pH = 7. The reactions of conversion between $[{\text{CO}}_{2} ]$ and $[{\text{HCO}}_{3}^{ - } ]$ $[{\text{H}}_{2 } {\text{S}}]$ and $[{\text{HS}}^{ - } ]$ and the corresponding dissociation constants *K*_1_ and *K*_2_ are as follows:

24 $$\left[ {{\text{CO}}_{2 } } \right] + \left[ {{\text{H}}_{ 2} {\text{O}}} \right] \rightleftarrows \left[ {{\text{H}}^{ + } } \right] + \left[ {{\text{HCO}}_{3}^{ - } } \right]$$

25 $$\left[ {{\text{H}}_{2 } {\text{S}}} \right] \rightleftarrows \left[ {{\text{H}}^{ + } } \right] + \left[ {{\text{HS}}^{ - } } \right]$$

26 $$K_{1} = \frac{{\left[ {{\text{H}}^{ + } } \right]\left[ {{\text{HCO}}_{3}^{ - } } \right]}}{{\left[ {{\text{CO}}_{2} } \right]}}$$

27 $$K_{2} = \frac{{\left[ {{\text{H}}^{ + } } \right]\left[ {{\text{HS}}^{ - } } \right]}}{{\left[ {{\text{H}}_{2} {\text{S}}} \right]}}$$

The constants used in the model are presented in Table 1.

**Table 1:** The constants used in the model

Constant	Values
$K_{1}$	1.37 × 10^−6^ mol/kg
$K_{2}$	2.2 × 10^−7^ mol/kg
$K{\text{h}}_{2}$	7.1 × 10^−9^ mol/L/Pa
$K_{{{\text{CO}}_{2} }}$	1.33 × 10^−7^ mol/L/Pa
${\text{Kh}}_{2} {\text{s}}$	2.1 × 10^−7^ mol/L/Pa
$D_{{{\text{O}}_{2} }}$	0.46 × 10^−4^ cm^2^/s
$D_{{{\text{H}}_{2} }}$	1.4 × 10^−5^ cm^2^/s
$D_{{{\text{CO}}_{2} }}$	0.66 × 10^−4^ cm^2^/s
$D_{{{\text{H}}_{2} {\text{S}}}}$	0.46 × 10^−4^ cm^2^/s
${\text{pH}}$	7
$R$	8.314 × 10^3^ Pa L/mol°K
$T$	80 °C
$V_{\text{g}}$	0.5 L
$V_{\text{l}}$	1.5 L

## Results and discussion

### Determination of kinetic and mass transfer parameters

#### Yields and kinetic parameters

To determine *μ*_max_, *μ*_d_,$Y_{{{\text{X}}/{\text{GLU}}}} , \;Y_{{{\text{X}}/{\text{YEAST}}}}$, $Y_{{{\text{X}}/{\text{THIO}}}}$, $K_{\text{sglu}} ,\;K_{\text{syeast}} \;{\text{and}},\;K_{\text{sthio}}$ calculations were carried out with experimental data from batch fermentations (2-L bioreactor). All these parameters are listed in Table 2.

**Table 2:** Parameters of the model

$\mu_{\rm{max} }$ (h^−1^)	$\mu_{\text{d }}$ (h^−1^)	$m_{\text{GLU}}$** mmol/g/h	$Y_{{{\text{X}}/{\text{GLU}}^{\text{a}} }}$ (g/mol)	$Y_{{{\text{X}}/{\text{YEAST}}}}$ (g/g)	$Y_{\text{X/THIO}}$ * (g/mmol)	$K_{\text{sglu}}$ mmol/L	$K_{\text{syeast}}$ g/L	$K_{\text{sthio}}$ mmol/L	$\left[ {{\text{H}}_{{ 2 {\text{crit}}}} } \right]$ mmol/L	*N*
0.9 ± 0.05	0.05 ± 0.02	2.2	20.9 ± 3.2	0.67 ± 0.11	3.617 ± 0.18	5.7 ± 1.1	0.30 ± 0.1	0.052 ± 0.01	1.44 ± 0.01	1

* Boileau et al. [part I, 21]

** Rinker et Kelly [23]. This value of $m_{\text{GLU}}$ was obtained for *T. maritima* culture in chemostat

^a^ $Y_{{{\text{X}}/{\text{GLU}}}}$ used for the mathematical model was of 20.9 g/mol. This value was obtained by subtracting the quantity of glucose used for the maintenance. The following values were used: $Y_{{{\text{X}}/{\text{GLU}}}}$ = 20.4 g/mol, $m_{\text{GLU}}$ = 2.2 mmol/g/h and *μ* = *μ*_max_ = 0.9 h^−1^

$\mu_{ \rm{max} }$ and *μ*_d_ were obtained for yeast extract, thiosulfate, and glucose concentrations of 4 g/L, 0.12, and 60 mmol/L, respectively with a stripping rate of 100 mL/min. The maximum growth rate of *T. maritima* ($\mu_{max }$ = 0.7 − 0.9 h^−1^), measured in this study, was comparable to those obtained by Huber et al. [20] for *T. maritima* (0.6 h^−1^) and, *T. naphthophila* (0.7 h^−1^) and *T. petrophila* (0.77 h^−1^), two species very closely related to *T. maritima* [13]. Since, in these experiments, the conditions were meant to allow obtaining the highest possible $\mu_{{{ \rm{max} } }}$, and in the present model, $\mu_{{{ \rm{max} } }}$ was chosen at 0.9 h^−1^. This value is higher than the ones obtained with *C. saccharolyticus* (0.2–0.5 h^−1^), an extreme thermophile H_2_-producing bacterium among the most studied [3, 24, 25]. This fairly high growth rate is an advantage for this H_2_ producer that allows the reduction of the H_2_-production time. For our model, the average value of $\mu_{\text{d}}$, obtained from experiments, was estimated at 0.05 h^−1^. Very few values of cell death rate ($\mu_{\text{d}}$) of hyperthermophilic microorganisms are available in the literature. This parameter ranges from 0.014 to 0.105 h^−1^ [3, unpublished data].

**Fig. 1:**
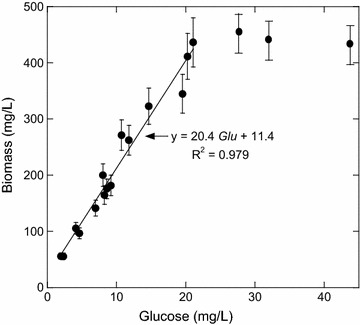
Effect of glucose concentration on *T. maritima* growth cultivated in presence of thiosulfate (0.12 mmol/L) and yeast extract (1 g/L). These cultures were performed in triplicate in bioreactor. Total biomass yield on glucose (20.4 g biomass/mol glucose) was evaluated from a linear regression ($20.4\;{\text{Glu}} + 11.4$)

$Y_{{{\text{X}}/{\text{GLU}}}}$ and $K_{\text{sglu}}$ were evaluated in batch cultures for a range of glucose concentrations between 0.3 and 60 mmol/L with thiosulfate and yeast concentrations of 0.12 mmol/L and 1 g/L, respectively. Figure 1 represents biomass content versus glucose concentration and shows that below 20 mmol/L, glucose was the sole nutritional factor limiting *T. maritima* growth. Then total biomass yield on glucose (20.4 g biomass/mol glucose) was evaluated from a linear regression ($20.4\;{\text{Glu}} + 11.4$) in this first part. The true $Y_{{{\text{X}}/{\text{GLU}}}}$ was determined from this total biomass yield after subtracting, from the quantity of glucose consumed, the part used for the maintenance. A resulting value of 20.9 g biomass/mol glucose was obtained by taking into account the maintenance coefficient (2.2 mmol/g/h) and $\mu_{ \rm{max} }$(0.9 h^−1^) (Table 2). $Y_{{{\text{X}}/{\text{s}}}}$ is comparable to those previously determined in batch or chemostat for *Thermotogales* and *Thermococcales* [6]. These values ranged from 13 to 45 g biomass/mol glucose. From growth kinetic data, $K_{\text{sglu}}$ was estimated at 5.7 mmol/L (Additional file 1: Fig. S1). This value of $K_{\text{sglu}}$ was much higher than the one obtained by Rinker and Kelly [23] (0.015 mmol/L) for *T. maritima* culture in chemostat. This difference may be linked to the fact that all values of *K*_s_ were determined in reactor batch cultures and did not represent a *K*_s_ per sé but an apparent *K*_s_. From now on, the constant *K*_s_ will in reality correspond to *K*_s,app_.

**Fig. 2:**
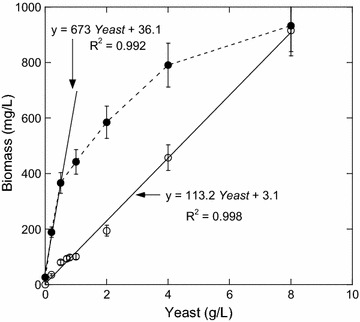
Effect of yeast concentration on *T. maritima* growth cultivated with glucose (60 mmol/L) and in the presence (0.12 mmol/L) or absence of thiosulfate. (*filled circle*) Maximal biomass obtained in presence of thiosulfate. (*open circle*) Maximal biomass obtained in absence of thiosulfate. These cultures were performed in triplicate in bioreactor. Biomass yield on yeast extract (0.673 g biomass/g yeast extract) was evaluated from a linear regression ($673\; {\text{Yeast}} + 36.1$). The linear regression $(113.2\;{\text{Yeast}} + 3.1)$ was obtained in absence of thiosulfate

$Y_{{{\text{X}}/{\text{YEAST}}}}$ and $K_{\text{syeast}}$ were determined for different concentrations of yeast extract (0.2, 0.5, 1, 2, 4, and 8 g/L) with thiosulfate concentrations of 0 or 0.12 mmol/L and glucose concentration of 60 mmol/L. Effect of yeast extract concentration on *T. maritima* growth is presented in Fig. 2. Without thiosulfate and up to 8 g/L of yeast extract, maximum biomass is always lower than when 0.12 mmol/L of thiosulfate was added. For this range of yeast extract concentrations, thiosulfate is the growth-limiting factor of *T. maritima* confirming the significant effect of thiosulfate addition on cell mass growth [part I, 21]. Moreover, when 0.12 mmol/L of thiosulfate is added and below 0.5 g/L of yeast extract, thiosulfate is the only growth-limiting factor (Fig. 2). Therefore, $Y_{{{\text{X}}/{\text{YEAST}}}}$ was determined in this window and was equal to 0.67 g biomasse/g YE (Table 2). It is noteworthy that $Y_{{{\text{X}}/{\text{YEAST}}}}$ decreases to 0.11 g/g in absence of thiosulfate (Fig. 2) showing that $Y_{{{\text{X}}/{\text{YEAST}}}}$ depends on the thiosulfate concentration. However, due to the lack of data between 0 and 0.12 mmol/L of thiosulfate, no relation of $Y_{{{\text{X}}/{\text{YEAST}}}}$ versus thiosulfate could be established. For the following of this study, $Y_{\text{X/YEAST}} \;{\text{was}}\;{\text{considered }}$ constant and equal to 0.67 g/g. $K_{\text{syeast}}$ of 0.3 g/L was obtained in this study from batch experiments (Additional file 1: Fig. S2)

$Y_{{{\text{X}}/{\text{THIO}}}}$ of 3.167 g/mmol was obtained by Boileau et al. [part I, 21] from batch culture experiments. $Y_{{{\text{X}}/{\text{THIO}}}}^{\text{X}}$(Eq. 8) represents the equivalent sulfur of the thiosulfate incorporated into the biomass and was calculated from the elemental composition of *T. maritima* [23]. It is equal to 10.47 g biomass/mmol thiosulfate. $Y_{\text{X/THIO}}^{{{\text{H}}_{2} {\text{S}}}}$ was determined from the difference between the total yield of thiosulfate $Y_{\text{X/THIO}}$ and $Y_{\text{X/THIO}}^{\text{X}}$, considering that all sulfur not incorporated into the biomass was reduced into H_2_S (Eq. 2). $Y_{{{\text{X}}/{\text{THIO}}}}^{{{\text{H}}_{2} {\text{S}}}}$ was equal to 5.52 g biomass/mmol thiosulfate. $K_{\text{sthio}}$, obtained in this study from batch experiments, was of 0.052 mmol/L (Additional file 1: Fig. S3).

In this study, two sulfur sources for *T. maritima* growth are available: thiosulfate added to the culture medium and sulfur (cysteine, etc…) in the yeast extract. In the previous paper [part I, 21], the authors determined an equivalent thiosulfate concentration of 0.03 mmol per g of yeast extract ($\varepsilon_{2}$, Eq. 7). A similar approach was used to determine the equivalent content of glucose in 1 g of yeast extract (Fig. 1). This value was obtained using the linear regression $X = 20.4\;{\text{Glu}} + 11.4$ by extrapolating the line to a cell mass concentration ($X$) equal to 0. Therefore, the equivalent glucose per g of yeast extract was of 0.56 mmol ($\varepsilon_{1}$, Eq. 5).

#### Determination of $Y_{\text{ACT/GLU}}$ and [H_2crit_] the critical dissolved-H_2_ concentration

**Fig. 3:**
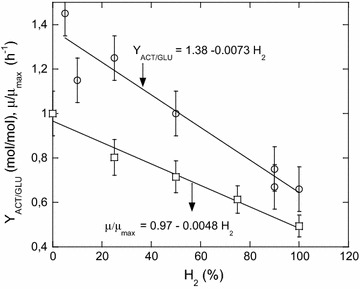
Acetate yield $Y_{{{\text{ACT}}/{\text{GLU}}}}$ (*open circle*) and specific growth rate (*μ/μ*_max_) (*open square*) as a function of hydrogen percentage in the gas phase. Linear regression for both graphs are indicated within the Figures. It shows $Y_{{{\text{ACT}}/{\text{GLU}}}}$ and *μ/μ*_max_ decrease of about 50% when percentage of hydrogen in the gas phase increase from 0 to 100%

$Y_{\text{ACT/GLU}}$ (stoichiometric parameter *a*, Eq. 1) was determined with different percentages of inlet H_2_ (0, 5, 10, 25, 50, 75, and 100%), for yeast extract, thiosulfate, and glucose concentrations of 4 g/L, 0.12, and 60 mmol/L, respectively. The stripping rate was 100 mL/min. Fig. 3 shows that up to 100% of H_2_, a linear relation of $Y_{\text{ACT/GLU}}$ versus the percentage of H_2_ in the gas phase was observed. By linear extrapolation ($Y_{{{\text{ACT}}/{\text{GLU}}}}$ = 0), critical H_2_ percentage in the gas phase was evaluated at 190%, above the value of the expected 100%. In the same way, the linear relation of *μ/μ*_max_ versus the percentage of H_2_ in the gas phase can be extrapolated (*μ/μ*_max_ = 0), and leads to a critical H_2_ percentage in the gas phase at 206%. For these two extrapolated H_2_ percentages in the gas phase, the critical dissolved hydrogen concentrations ($\left[ {{\text{H}}_{{2{\text{crit}}}} } \right]^{*}$) in equilibrium with the critical H_2_ concentrations in the gas phase are of 1.33 and 1.44 mmol/L, respectively. $\left[ {{\text{H}}_{{ 2 {\text{crit}}}} } \right]^{*}$ has been evaluated, and in the same way, the true critical dissolved hydrogen ($\left[ {{\text{H}}_{{ 2 {\text{crit}}}} } \right]$) cannot be measured. However, for the theoretical case where *μ*/*μ*_max_ and $Y_{\text{ACT/GLU}}$ are both equal to 0, no gradient of H_2_ in the liquid phase takes place and consequently $\left[ {{\text{H}}_{{2{\text{crit}}}} } \right]$ is equal to $\left[ {{\text{H}}_{{ 2 {\text{crit}}}} } \right]^{*}$. It is noteworthy that the estimation of $\left[ {{\text{H}}_{{2{\text{crit}}}} } \right]$ (1.3–2.2 mmol/L) obtained from a kinetic model of the effect of hydrogen and osmolarity on hydrogen production by *C. saccharolyticus* [3] were coherent with our estimates (1.3–1.4 mmol/L).

$Y_{\text{ACT/GLU}}^{ \rm{max} }$ was obtained from the experimental correlations for ${\text{H}}_{2}$ = 0% (Fig. 3). It is equal to 1.38 mol/mol. $Y_{{{\text{H}}_{2} /{\text{GLU}}}}^{ \rm{max} }$ and $Y_{{{\text{CO}}_{2} /{\text{GLU}}}}^{ \rm{max} }$ were deduced from $Y_{{{\text{ACT}}/{\text{GLU}}}}^{ \rm{max} }$ using the Eq. 1. Thus, $Y_{{{\text{H}}_{2} /{\text{GLU}}}}^{ \rm{max} }$ and $Y_{{{\text{CO}}_{2} /{\text{GLU}}}}^{ \rm{max} }$ are equal to 2.76 and 1.38 mol/mol, respectively. Mars et al. [16] measured similar values of $Y_{\text{ACT/GLU}}^{ \rm{max} } \;\left( {1.4\;{\text{mol}}/{\text{mol }}} \right),\; Y_{{{\text{H}}_{2} /{\text{GLU}}}}^{ \rm{max} }$ (2.9 mol/mol), and $Y_{{{\text{CO}}_{2} /{\text{GLU}}}}^{ \rm{max} }$ (1.6 mol/mol) for *T. neapolitina* growing on glucose in batch culture. Values of $Y_{{{\text{H}}_{2} {\text{S}}/{\text{THIO}}}}$ and $Y_{{{\text{H}}_{2} /{\text{H}}_{2} {\text{S}}}}$ were deduced using the Eq. 2. Both values are equal to 2 mol/mol.

Because dissolved H_2_ ($\left[ {{\text{H}}_{2} } \right]$) cannot be measured, the parameter *N* (Eq. 4) cannot be directly evaluated. Datasets from five experiments were used to evaluate the maximum of H_2_ productivity, which was compared to the maximum H_2_ productivity obtained from the mathematical model for *N* values of 0.5, 1, and 1.5. For these experiments, at least one substrate (thiosulfate, glucose, or yeast extract) is the growth-limiting factor [part I, 21]. For each experimental condition, the average of the difference between the maximum of H_2_ productivity obtained from the model and from the experiment was -2.2, 0.2, and 1.5 mmol/L/h for *N* equal to 0.5, 1, and 1.5, respectively. Ljunggren et al. [3] estimated, using another approach with *C. saccharolyticus*, the parameter *N*, from experiments with 5 g/L of glucose and different stripping rates, ranged from 20 to 100 mL/min. These authors obtained a mean value of *N* (4.5) with a large standard deviation (0.84–16.24). For the rest of our study, a value of *N* equal to 1 was chosen.

#### Determination of the volumetric mass transfer coefficient

Since H_2_, CO_2_, and H_2_S are sparingly soluble gases, the gas phase mass transfer resistance can almost always be neglected [26]. Thereby, the overall volumetric mass transfer coefficient, ${\text{Kla}}$, is adequate for describing the mass transfer. Assuming that the mass transfer coefficients of the different gases in the water are proportional to the square root of their diffusivity, the ${\text{Kla}}$ values of H_2_, CO_2_, and H_2_S can be calculated on the basis of the experimental data obtained from the determination of the ${\text{Kla}}$ O_2_ (Eq. 29).

28 $${\text{KlaG}} = {\text{KlaO}}_{2} \left( {\frac{{D_{\text{G}} }}{{D_{{{\text{O}}_{2} }} }}} \right)^{0.5} \quad{\text{with}}\;{\text{G}} = {\text{H}}_{ 2} ,\;{\text{CO}}_{ 2} \;{\text{or}}\;{\text{H}}_{ 2} {\text{S}}$$

$D_{{{\text{G}} = {\text{H}}_{2} ,\;{\text{CO}}_{2} ,\;{\text{H}}_{2} {\text{S}}}}$ are the H_2_, CO_2_, and H_2_S coefficients of diffusion in water at 80 °C (Table 1).

${\text{KlaO}}_{2}$ was determined by measuring the dissolved O_2_ concentration in the sterile culture medium [27]. It was obtained in the 2-L bioreactor by either physical absorption or desorption of oxygen. Air-flow rate was tested in a range from 20 to 500 mL/min at a constant-speed agitation (350 rpm).

The correlation between ${\text{KlaO}}_{2}$ and $Q_{{{\text{N}}_{2} }}$ is as follows:

29 $${\text{KlaO}}_{2} = 18.11 \;Q_{{{\text{N}}_{2} }}^{0.721} \quad{\text{with}}\;Q_{{{\text{N}}_{2} }} \;{\text{in}}\;{\text{L/h}}$$

From the Eqs. 28 and 29, the ${\text{KlaH}}_{2}$ was of 114 h^−1^ for a $Q_{{{\text{N}}_{2} }}$ of 100 mL/min and a stirring rate of 350 rpm. This value is significantly higher than those usually reported in the literature for similar systems [3, 28]. Ljunggren et al. [3] measured a ${\text{KlaH}}_{2}$ value of 9 h^−1^ at 70 °C for the same $Q_{{{\text{N}}_{ 2} }}$ and stirring rate. A ${\text{KlaH}}_{2}$ of 17 h^−1^ was obtained for $Q_{{{\text{N}}_{ 2} }}$ of 100 mL/min, but for lower temperature (35 °C) and with an unknown value of stirring rate (use of a stir plate) [28]. In our study, the higher ${\text{KlaH}}_{2}$ could be explained by the following: (1) the high-temperature experiments (80 °C) increasing the diffusion coefficient of H_2_ in water, (2) the use of a 3-cm-long fritted cylinder gas-dispersion stone with 4–60 μM pore size allowing the formation of numerous very small bubbles of gas and, (3) the use of two axial impellers promoting an effective mixing. ${\text{KlaCO}}_{2}$ and ${\text{KlaH}}_{2} {\text{S}}$ were determined in the same way as ${\text{KlaH}}_{2}$.

### Model validation

The mathematical model developed in this study must be able to both provide new knowledge and predict the optimal operating conditions of the H_2_ production by *T. maritima*. For this, the mathematical model was validated for various glucose, yeast extract, and thiosulfate concentrations and inlet N_2_ flow rates. Forty experiments were carried out with different concentration ranges of glucose (2.5–63 mmol/L), yeast extract (0.2–8 g/L), thiosulfate (0.01–2 mM), and inlet N_2_ flow rates (17–190 mL/min). These various operating conditions correspond to situations where one or none of these compounds (glucose, yeast extract, and thiosulfate) is the limiting-growth factor.

**Fig. 4:**
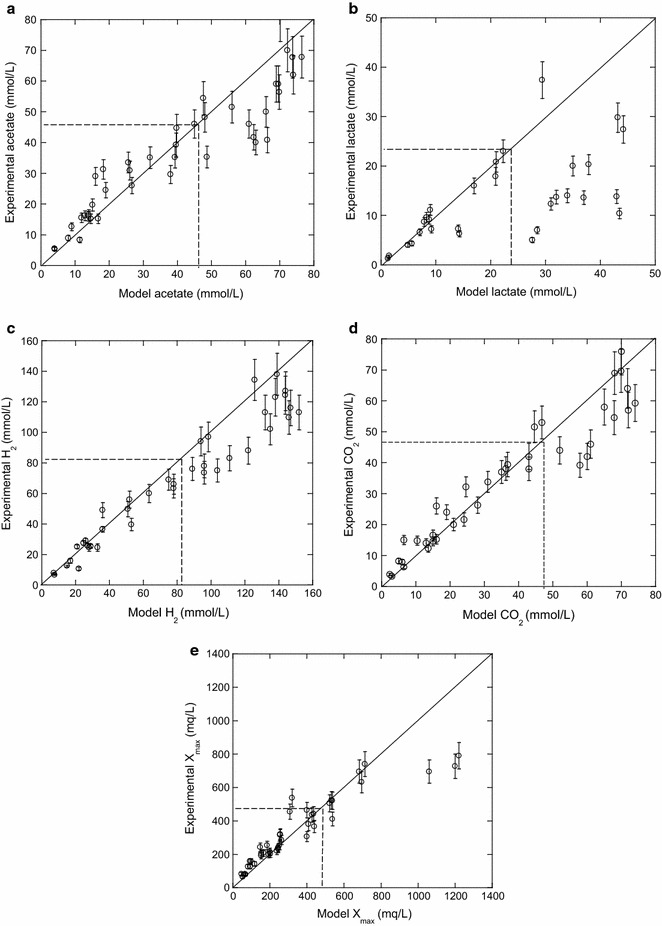
Comparison of experimental and model end-product productions for glucose (2.5–63 mmol/L), yeast extract (0.2–8 g/L), thiosulfate (0.01–2 mmol/L) and an inlet N_2_ flow rates between 17 and 190 mL/min. **a** Acetate production, **b** lactate production, **c** H_2_ production, **d** CO_2_ production, and **e** maximum of biomass. *Dashed lines* correspond to the conditions above which neither glucose nor yeast extract nor thiosulfate is the limiting factor. Beyond this limit, except for lactate, the model overestimates all the variables of about 30%

Figure 4a–e represents the comparison between the experimental and model results for acetate, lactate, H_2_ and CO_2_ productions, and the maximum of biomass, respectively. It is noteworthy that, for the five comparisons, a same pattern can be observed. When one of the compounds of interest is at a concentration for which it is the limiting-growth factor, a good correlation between the experiment and the model is noted. On the contrary, when all the concentrations are above the limiting factor level, the model overestimates the productions. The mathematical model is adequate for glucose, yeast extract, and thiosulfate concentrations ranging from 2.5 to 20 mmol/L, 0.2–0.5 g/L, and 0.01–0.06 mmol/L, respectively. As expected, these ranges correspond to the value for which $Y_{{{\text{X}}/{\text{GLU}}}} ,\;Y_{{{\text{X}}/{\text{YEAST}}}} ,$ $Y_{{{\text{X}}/{\text{THIO}}}}$ were determined experimentally (Table 2). When glucose, yeast extract, and thiosulfate concentrations are all higher than these ranges, they are beyond the limit for which the yields of biomass on glucose, yeast extract, and thiosulfate were determined (Figs. 1, 2, and part I [21]). It is therefore logical that the model will overestimate all the variables (acetate, lactate, H_2_, and CO_2_ productions and, maximum of biomass) (Fig. 4a–e). This overestimation is about between 10 and 50%, except for lactate with a percentage close to 100% (Fig. 4b). This overestimation could be due to the fact that one or more of unknown variables are inhibiting and/or limiting the growth of *T. maritima* and more studies are needed to identify them.

**Fig. 5:**
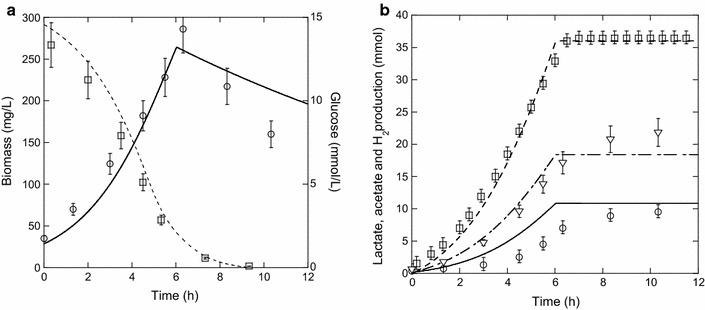
**a**, **b** Experimental and model results for glucose (14 mmol/L), yeast extract (1 g/L), thiosulfate (0.12 mmol/L), and an inlet N_2_ flow rates of 100 mL/min. Experimental results: **a** biomass (*open circle*) and glucose (*open square*), **b** H_2_ (*open square*), acetate (*inverted triangle*), and lactate (*open circle*) Model results: **a** biomass (*solid lines*) and glucose (*dotted lines*), (**b**) H_2_ (*dotted lines*), acetate (*dashed–dotted lines*) and lactate (*solid lines*)

Figure 5a, b represents an example of experimental and model results for the following operating conditions: ${\text{Glu }}$ = 14 mmol/L, ${\text{Yeast}}$ = 1 g/L, ${\text{Thio}}$ = 0.12 mmol/L, and $Q_{{{\text{N}}_{ 2} }}$ = 100 mL/min. In these conditions, glucose is the limiting-growth factor. After 6 h of fermentation, the maximum of biomass (*X*_max_ = 290 ± 30 mg/L) was attained, and 80% of the glucose was consumed (Fig. 5a). H_2_ productivity (7 mmol/L h) reached the maximum after 5 h and slightly decreased until 9 h (data not shown) corresponding to the time when the glucose was totally consumed. A clear maintenance phase was observed between 6 and 9 h (Fig. 5a). During this time, the residual glucose is consumed, with a strong decrease in its consumption rate, and this consumption is not associated with biomass increase. However, in the meantime, 10% or more of the total H_2_ and acetate were produced, and a partial metabolic shift from acetate toward lactate was observed (Fig. 5b).

In the model, equations related to sulfur have been taken into account. To experimentally confirm the model, the thiosulfate disappearance needs to be monitored. In order to do so, experiments with increased thiosulfate concentration to 20 mmol/L were performed (Glucose 60 mmol/L, Yeast extract 4 g/L, and $Q_{{{\text{N}}_{ 2} }}$ 100 mL/min). In this experiment, none of the compounds of interest was the limiting factor for growth. In this case, 4 mmol/L of thiosulfate were consumed and the total H_2_S production measured at the end of the fermentation was of 3.5 mmol. Similar maximum of biomass and H_2_ productivity, acetate and H_2_ productions were obtained in the same conditions with 0.12 mmol/L thiosulfate (data not shown), corroborating that thiosulfate was not the growth-limiting and or -inhibiting factor. The addition of 4 g/L of yeast extract increases the equivalent initial total thiosulfate concentration (${\text{Thio}} + \varepsilon_{2}$) from 20 to 20.24 mmol/L. A negligible part of this thiosulfate (0.07 mmol/L) is incorporated as sulfur into the biomass $\left( {{\text{CH}}_{1.6} {\text{O}}_{0.6} {\text{N}}_{0.2} {\text{S}}_{0.005} } \right)$ [23], the remaining consumed thiosulfate (3.93 mmol/L) should be converted into H_2_S (7.86 mmol/L, Eq. 2). However, the total H_2_S production measured experimentally (3.5 mmol) is about 2 times lower, showing that not all the thiosulfate was converted into H_2_S and that probably some other unknown sulfur compounds are produced during the fermentation.

### Prediction of the mathematical model

In this study, a mathematical model of the fermentation of *T. maritima* has been written. This model has been proved to be in agreement with the experiment in a certain range of concentrations of glucose, yeast extract, and thiosulfate, and of the inlet gas (N_2_) flow rate. The final purpose of this model is to provide, among other things, a mean of predicting specific H_2_ productivity linked to H_2_ inhibition for *T. maritima* in various situations.

Studies have showed that a decrease of N_2_ stripping rate (i.e., mass transfer coefficient, KlaH_2_) resulted in a lower productivity and yield of H_2_ [3]. Our results showed that between 100 and 180 mL/min, no change of experimental maximum H_2_ productivity and glucose consumption rate was observed, while a strong decrease (about 50%) was recorded when $Q_{{{\text{N}}_{2} }}$ was reduced to 20 mL/min (Additional file 1: Fig. S4). The model was used to simulate the specific H_2_ productivity and the inhibition by H_2_ versus the N_2_ inlet flow rate ($Q_{{{\text{N}}_{2} }}$, 5–100 mL/min) for different operating conditions (0 < Gluc < 20 mmol/L or 0.1 < Yeast < 0.5 g/L or 0 < Thio < 0.06 mmol/L). These ranges correspond to the concentrations where the model was accurately validated (Fig. 4a–e). Initial biomass concentration, volume of liquid of bioreactor, and volume of gas (headspace) were set to 31.6 mg/L, 1.5, and 0.5 L, respectively.

**Fig. 6:**
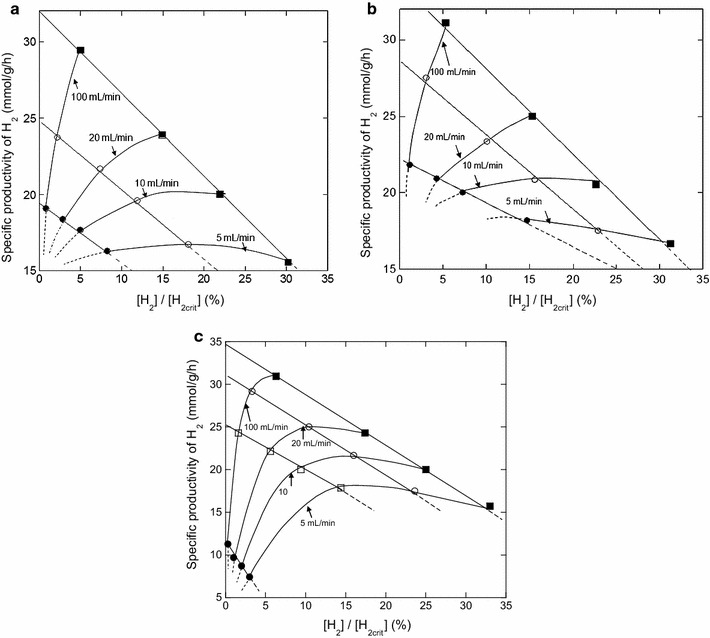
**a**–**c** Specific H_2_ productivity versus the ratio of the maximum dissolved-H_2_ concentration on the critical dissolved-H_2_ concentration $\left( {\left[ {{\text{H}}_{2} } \right] /\left[ {{\text{H}}_{{ 2 {\text{crit}}}} } \right]} \right)$ predicted by the mathematical model at various inlet N_2_ flow rates (5, 10, 20, and 100 mL/min). **a** For three yeast extract concentrations: (*filled circle*) 0.1 g/L, (*open circle*) 0.25 g/L, and (*filled square box*) 0.5 g/L. Glucose and thiosulfate concentrations were 50 and 0.12 mmol/L, respectively. **b** For three thiosulfate concentrations: (*filled circle*) 0 mmol/L, (*open circle*) 0.03 mmol/L, and (*filled square box*) 0.06 mmol/L. Glucose and yeast extract concentrations were 50 mmol/L and 1 g/L, respectively. **c** For four glucose concentrations: (*filled circle*) 0 mmol/L, (*filled square*) 5 mmol/L, (*open circle*) 10 mmol/L, and (*filled square box*) 20 mmol/L. Thiosulfate and yeast extract concentrations were 0.12 mmol/L and 1 g/L, respectively. For these three model predictions one of the variables (glucose, yeast extract, and thiosulfate concentrations) is growth-limiting

Figure 6a–c represents the maximum specific H_2_ productivity against the ratio of the maximum dissolved-H_2_ concentration on the critical dissolved-H_2_ concentration $\left( {\left[ {{\text{H}}_{ 2} } \right] /\left[ {{\text{H}}_{{ 2 {\text{crit}}}} } \right]} \right)$ for various $Q_{{{\text{N}}_{2} }}$. It appears that inhibition by H_2_, formalized by $\left[ {{\text{H}}_{2} } \right]/\left[ {{\text{H}}_{{ 2 {\text{crit}}}} } \right]$, never exceeds 33% (Fig. 6a–c) which corresponds to 11.1 mmol/L of H_2_ concentration in the gas phase. Such concentration (11.1 mmol/L) corresponds to a maximum dissolved H_2_ of 0.475 mmol/L, showing that, for these operating conditions, reaching the critical H_2_ dissolved concentration (1.44 mmol/L), for which *T. maritima* growth is stopped, seems impossible. Moreover, for this inhibition by H_2_, yield of hydrogen is little affected, it decreases from 2.76 to 2.3 mol/mol.

In almost all the cases, the increase in concentration of the limiting factor leads to a notable increase in specific H_2_ productivity. However, this increase, when the produced H_2_ is slightly flushed by the inlet gas stream (5 mL/min), leads to a strong relative inhibition. Whatever the operating conditions, when N_2_ stripping rate increases from 5 to 100 mL/min, the inhibition by H_2_ strongly decreases from 33 to 5% (Fig. 6a–c). The combination of both variables (increase in limiting factor concentration and in inlet gas stream) leads up to a twofold increase of the maximum H_2_ specific productivity with the lowest inhibition.

This model can be extended beyond the upper limits for non-limiting concentrations of glucose (>20 mmol/L), thiosulfate (>0.06 mmol/L), and yeast extract (0.5 g/L). However, in these cases, the mathematical model will overestimate the production of hydrogen of about 30% (Fig. 4c). Moreover, this model cannot be validated without sparging or for low stripping rates (<5 mL/min). Indeed, for these conditions, gas-transfer diffusion (H_2_, CO_2_, N_2_, and H_2_S) becomes predominant. This parameter is not taken into account in our model. In this case, H_2_ inhibition will be important and will affect strongly the specific H_2_ productivity.

## Conclusions

Batch fermentations of *T. maritima* were successfully simulated using a mathematical model that incorporates the kinetics of growth, consumptions of substrates (glucose, yeast extract, and thiosulfate) and product formations (H_2_, CO_2_, H_2_S, acetate, and lactate). Except for one, all of the model parameters were determined experimentally. However, the limits of the validity of this model were clearly established, and it is within the ranges when glucose or yeast extract or thiosulfate limit the growth. Anyway, the development of structured (mechanistic) models for quantifying microbial growth kinetics are still limited because the mechanism of cell growth is very complex and is not yet completely understood. Moreover, the defined mineral media are formulated so as to allow microorganisms to synthesize their cellular components from single sources of carbon, sulfur, ammonium, phosphorus… Usually, only one of them limits the maximum quantity of biomass that could be produced, with all other nutrient in excess. We focused our interest on four state variables (glucose, yeast extract, thiosulfate, and hydrogen concentrations), but we should not dismiss the potential other limiting factors present in the medium. In the future, it would be interesting to research other potential limiting factors and to take into account their influence on the model. For example, Rinker and Kelly [23] demonstrated that the lower NH_4_Cl concentration (0.5 g/L our study) would be limiting for the growth of *T. maritima*.

From now on, this model can be use, in the limits of its validity, to predict, depending on the scenario, many elements, and in particular, specific H_2_ productivity, dissolved-H_2_ concentration, etc.

## Additional file

**Additional file 1: Figure S1.** Determination of *K*_*sgluc*_. m1 = *K*_*sgluc*_, M0: Glucose concentration, m2 = *μ*_max_. **Figure S2.** Determination of *K*_*syeast*_. m1 = *K*_*syeast*_, M0: Yeast extract concentration, m2 = *μ*_max_. **Figure S3.** Determination of *K*_*sthio*_. m1 = *K*_*sthio*_, M0: Thiosulfate concentration, m2 = *μ*_max_. $K_{sthio,} K_{syeast}$ and $K_{sglu }$ were estimated by fitting Monod’s equation to experimental data. **Figure S4.** Experimental maximum volumetric H_2_ productivity and glucose consumption rate versus *Q*_*N2*_.

## Abbreviations

${\text{Act}}$: acetate concentration (mol/L)
[CO_2_]: CO_2_ concentration in the liquid phase (mol/L)
$\left[ {{\text{CO}}_{2} } \right]^{*}$: concentration of dissolved CO_2_ at thermodynamic equilibrium (mol/L)
${\text{D}}_{{{\text{O}}_{2} }}$: coefficient of diffusion in water for O_2_ (cm^2^/s)
$D_{{{\text{H}}_{2} }}$: coefficient of diffusion in water for H_2_ (cm^2^/s)
$D_{{{\text{CO}}_{2}}}$: coefficient of diffusion in water for CO_2_ (cm^2^/s)
$D_{{{\text{H}}_{2}{\text{S}}}}$: coefficient of diffusion in water for H_2_S (cm^2^/s)
${\text{Glu}}$: glucose concentration (mol/L)
H_2_: H_2_ concentration in the gas phase (mol/L)
$\left[ {{\text{H}}_{{2{\text{crit}}}} } \right]$: critical dissolved-H_2_ concentration (mol/L)
[H_2_]: H_2_ concentration in the liquid phase (mol/L)
[H_2_S]: H_2_S concentration in the liquid phase (mol/L)
[HCO_3_^−^]: ${\text{HCO}}_{3}^{ - }$ concentration in the liquid phase (mol/L)
$\left[ {{\text{HS}}^{ - } } \right]$: HS^−^concentration in the liquid phase (mol/L)
$\left[ {{\text{H}}_{2} } \right]^{*}$: concentration of dissolved H_2_ at thermodynamic equilibrium (mol/L)
[H_2_S]^*^: concentration of dissolved H_2_S at thermodynamic equilibrium (mol/L)
*K*_sglu_: saturation constant of glucose (mol/L)
*K*_syeast_: saturation constant of yeast extract (g/L)
*K*_sthio_: saturation constant of thiosulfate (mol/L)
KlaH_2_: volumetric mass transfer coefficient for H_2_ (h^−1^)
KlaCO_2_: volumetric mass transfer coefficient for CO_2_ (h^−1^)
KlaH_2_S: volumetric mass transfer coefficient for H_2_S (h^−1^)
$K_{1 }$: dissociation constant for CO_2_ into bicarbonate (mol/kg)
$K_{2 }$: dissociation constant for H_2_S into bisulfide (mol/kg)
Kh_2_: Henry’s constant for H_2_ (mol/L/Pa)
$K_{{CO_{2} }}$: Henry’s constant for CO_2_ (mol/L/Pa)
Kh_2_s: Henry’s constant for H_2_S (mol/L/Pa)
Lact: lactate concentration (mol/L)
$m_{\text{GLU}}$: maintenance coefficient (mol/g/h)
*N*: exponential parameter
$Q_{\text{g}}$: outlet-gas flow rate (L/h)
$Q_{{{\text{N}}_{ 2} }}$: inlet gas (N_2_) flow rate (L/h)
$Q_{{{\text{H}}_{2} }}$: outlet-gas (H_2_) flow rate (L/h)
${Q_{{\text{CO}}_{2}}}$: outlet-gas (CO_2_) flow rate (L/h)
$Q_{{{\text{H}}_{2} {\text{S}}}}$: outlet-gas (H_2_S) flow rate (L/h)
*R*: gas constant (Pa L/mol °K)
*T*: temperature (°K)
Thio: thiosulfate concentration
*V*_l_: free-liquid-space volume in the reactor (L)
*V*_g_: free-gaseous-space volume in the reactor (L)
*X*: cell mass concentration (g/L)
Yeast: yeast extract concentration (g/L)
*Y*_X/GLU_: biomass yield (g/mol)
*Y*_X/YEAST_: yeast extract yield (g/g)
*Y*_X/THIO_: thiosulfate yield (g/mol)
$Y_{{{\text{X}}/{\text{THIO}}}}^{\text{X}}$: thiosulfate yield (equivalent sulfur from thiosulfate incorporated into the biomass) (g/mol)
$Y_{{{\text{X}}/{\text{THIO}}}}^{{{\text{H}}_{2} {\text{S}}}}$: H_2_S yield (equivalent sulfur from thiosulfate released as H_2_S) (g/mol)
$Y_{{{\text{ACT}}/{\text{GLU}}}}$: acetate yield (mol/mol)
$Y_{{{\text{H}}_{2} /{\text{GLU}}}}$: H_2_ yield (mol/mol)
$Y_{{{\text{CO}}_{2} /{\text{GLU}}}}$: CO_2_ yield (mol/mol)
$Y_{{{\text{H}}_{2} /{\text{H}}_{2} {\text{S}}}}$: stoichiometric H_2_ on H_2_S yield (mol/mol)
$Y_{{{\text{H}}_{2} {\text{S}}/{\text{THIO}}}}$: stoichiometric H_2_S on thiosulfate yield (mol/mol)
$Y_{{{\text{ACT}}/{\text{GLU}}}}^{ \rm{max} }$: maximum yield of acetate (mol/mol)
$Y_{{{\text{H}}_{2} /{\text{GLU}}}}^{ \rm{max} }$: maximum yield of H_2_ (mol/mol)
$Y_{{{\text{CO}}_{2} /{\text{GLU}}}}^{ \rm{max} }$: maximum yield of CO_2_ (mol/mol)
$Y_{{{\text{H}}_{2} {\text{S}}/{\text{THIO}}}}$: stoichiometric H_2_S on thiosulfate yield (mol/mol)
$Y_{{{\text{H}}_{2} /{\text{H}}_{2} {\text{S}}}}$: stoichiometric H_2_ on H_2_S yield (mol/mol)
$\varepsilon_{1}$: equivalent concentration of glucose in the yeast extract (mol/L)
$\varepsilon_{2}$: equivalent concentration of thiosulfate in the yeast extract (mol/L)
*μ*: specific growth rate (h^−1^)
*μ*_max_: maximum specific growth rate (h^−1^)
