# Supplementary material for: Hydrogen production by the hyperthermophilic bacterium Thermotoga maritima Part II: modeling and experimental approaches for hydrogen production
Source: Biotechnol Biofuels. 2016 Dec 19;9:268. doi: 10.1186/s13068-016-0681-0 (PMC5168804; doi:10.1186/s13068-016-0681-0)
Supplement: Supplementary file 1 — Additional file 1: Figure S1. Determination of K sgluc. m1 = K sgluc, M0: Glucose concentration, m2 = μ max. Figure S2. Determination of K syeast. m1 = K syeast, M0: Yeast extract concentration, m2 = μ max. Figure S3. Determination of K sthio. m1 = K sthio, M0: Thiosulfate concentration, m2 = μ max. $K_{sthio,} K_{syeast}$Ksthio,Ksyeast and $K_{sglu }$Ksglu were estimated by fitting Monod’s equation to experimental data. Figure S4. Experimental maximum volumetric H2 productivity and glucose consumption rate versus Q N2. [file 13068_2016_681_MOESM1_ESM.docx]

**Additional files**

Figure 1': Determination of *K_sgluc_*_._ m1 = *K_sgluc_*_,_ M0: Glucose concentration_,_ m2 = μ_max_.

Figure 2': Determination of *K_syeast_*_._ m1 = *K_syeast_*_,_ M0: Yeast extract concentration_,_ m2 = μ_max_.

Figure 3': Determination of *K_sthio_*. m1 = *K_sthio_*_,_ M0: Thiosulfate concentration_,_ m2 = μ_max_.

$K_{sthio,} K_{syeast}$ and$K_{sglu}$were estimated by fitting Monod’s equation to experimental data.

Figure 4': Experimental maximum volumetric H_2_ productivity and glucose consumption rate versus *Q_N2_*.
